# Patterns of community violence exposure among urban adolescents and their associations with adjustment

**DOI:** 10.1002/ajcp.12598

**Published:** 2022-04-28

**Authors:** Sarah K. Pittman, Albert D. Farrell

**Affiliations:** ^1^ Department of Psychology Virginia Commonwealth University Richmond Virginia USA

**Keywords:** anxiety symptoms, Black/African American adolescents, community violence exposure, latent class analysis, physical aggression, urban adolescents

## Abstract

Community violence exposure is prevalent among urban and marginalized adolescents. Although there is strong evidence that community violence exposure is associated with negative consequences, prior studies and theories suggest that these associations may differ as a function of specific characteristics of exposure. This study identified patterns of community violence exposure that differed in form (witnessing vs. victimization), familiarity with the victim, and severity, and in their associations with adolescents' frequency of physical aggression and anxiety symptoms. Participants were 681 eighth‐grade adolescents (58% female, 95% African American). Latent class analysis identified five subgroups who reported distinct patterns of violence exposure: *limited exposure; witnessed less severe violence, not victimized; witnessed severe violence, not victimized; witnessed less severe violence, some victimization; and high violence exposure*. The *witnessed less severe, some victimization*, and *high violence exposure* subgroups reported the highest frequency of physical aggression and levels of anxiety compared with all other subgroups. The *limited exposure* subgroup reported the lowest frequencies of physical aggression. The findings suggest that the form of exposure (witnessing or victimization) is an important distinction in examining associations with adolescent adjustment. Limited support was found for differences related to familiarity with the victim and severity of violence.

## Highlights

- Nearly all adolescents reported community violence exposure, and their experiences varied widely.
- One subgroup had limited exposure, two only witnessed violence, and two experienced victimizations.
- The two victimized subgroups reported the highest levels of physical aggression and anxiety.
- Two subgroups who only witnessed violence had the second‐highest levels of physical aggression.
- Interventions could be tailored to youths' experiences to prevent consequences of exposure.

## INTRODUCTION

Youth who grow up in communities that have historically encountered structural inequalities and marginalization often experience a number of stressors that negatively impact their development (Foster & Brooks‐Gunn, 2009). For example, a recent study found that 35% of adolescents experienced at least one form of community violence in their lifetime (Chen et al., 2020). These rates are higher among adolescents of color residing in urban, historically underserved neighborhoods (54% for Black youth and 43% for Latinx youth) compared with White adolescents (22%) and adolescents growing up in suburban or rural settings (Chen et al., 2020). Adolescents exposed to community violence have a high risk of experiencing internalizing symptoms and emotional problems such as posttraumatic stress disorder (PTSD) and depression, and engaging in antisocial behavior such as aggression, fighting, and delinquency (Fowler et al., 2009). Moreover, the emotional and behavioral effects of exposure to community violence can lead to negative consequences, such as persistent mental illness, perpetration of violence, criminal activity, and poor school performance or school failure (Foster & Brooks‐Gunn, 2009).

Despite evidence that particular characteristics of exposure lead to differences in outcomes and may operate through different mechanisms (e.g., Kennedy & Ceballo, 2014), studies examining the causes and consequences of exposure to community violence among children and adolescents have been inconsistent in its measurement. Kennedy and Ceballo (2014) proposed that researchers examining the impact of exposure to violence on adolescents assess multiple characteristics of exposure (e.g., severity of exposure and form of violence). Nonetheless, many studies of the effects of exposure (e.g., Chen et al., 2020; Kennedy & Ceballo, 2016) have represented it by a single composite of items that encompass a variety of experiences. This approach assumes that all forms of exposure have an equal impact on adjustment (Kennedy & Ceballo, 2016). In contrast, person‐centered approaches (e.g., latent class analysis; LCA) can be used to identify subgroups that differ in their patterns across variables of interest. Thus, person‐centered approaches could be used to identify different patterns of adolescent experiences within their community to clarify the ways in which their environment (e.g., community violence) impacts adjustment. Understanding the ways in which nuances in characteristics of adolescents' experiences of community violence lead to differences in consequences is critical to refine theories and inform intervention efforts. Specific characteristics include the form (witnessing vs. experiencing victimization), degree of familiarity with the victim, and severity of violence. The goal of this study was to use LCA, which represents a person‐centered approach, to identify patterns of community violence exposure experienced by adolescents and their associations with adjustment.

### Characteristics of exposure

Although many previous studies have combined experiencing and witnessing violence into an overall composite score, a growing body of research suggests that experiencing and witnessing are distinct forms of exposure that differ in their consequences and in the underlying processes by which they influence adjustment. Studies employing factor analysis have indicated that community violence exposure is best represented by separate constructs that differentiate between witnessing and victimization (e.g., Martin et al., 2013; van Dulmen et al., 2008). The importance of this distinction is further supported by studies indicating that experiencing victimization is more strongly associated with internalizing symptoms compared with witnessing violence (for a review see Fowler et al., 2009). Although most studies have found that both witnessing community violence and victimization are positively associated with externalizing symptoms (Fowler et al., 2009), some studies have found differences in the strength of these associations. For example, a recent study of a predominately African American sample of urban adolescents found that witnessing community violence had unique effects on externalizing problems above and beyond the effects of victimization (Farrell et al., 2020). There is also evidence that different mechanisms might underly the associations between witnessing violence and experiencing victimization (e.g., Schwartz & Proctor, 2000). Prior studies have found that witnessing community violence increases normative beliefs about aggression and the use of violence (e.g., Farrell et al., 2021; Guerra et al., 2003), whereas victimization is thought to cause traumatic stress responses and difficulties with emotion regulation. This could explain why different forms of exposure differ in their associations with externalizing problems (Schwartz & Proctor, 2000). More research is needed to clarify the extent to which witnessing and victimization represent distinct constructs that differ in their associations with adjustment.

The effects of witnessing community violence may also vary depending on the observer's familiarity with the victim. Several studies have found that knowing the victim has a greater impact on an individual's internalizing symptoms (e.g., Lambert et al., 2012; Ward et al., 2001). Violence that occurs against someone with whom an adolescent is familiar versus a stranger might disrupt the adolescents' social network and prevent access to social support, which could increase their likelihood of developing internalizing symptoms (Lambert et al., 2012; Ward et al., 2001). It is also more likely to occur closer to where the adolescent lives (e.g., seeing a family member stabbed outside their house), which could compromise the sense of safety because of the physical proximity to the adolescent's home (Ward et al., 2001). Finally, witnessing violence against someone they know may increase adolescents' concerns that violence could happen to them, which could increase their risk of developing internalizing symptoms (Lambert et al., 2012). Thus, although there is evidence that the relationship with the victim of community violence is important, more work is needed to determine its effects on adolescent adjustment.

Research focusing on traumatic stress suggests that experiences involving threat to life or threat of serious injury have a greater influence on internalizing symptoms than less severe experiences. For example, witnessing an act of violence that involves a weapon (e.g., seeing someone stabbed) might be perceived by adolescents as more severe than an act of violence without a weapon, regardless of the severity of injury to the victim (Aisenberg et al., 2008). Findings from Goldner et al. (2015) suggest that associations between experiences of violence and internalizing and externalizing symptoms differ depending on the severity of the act (i.e., moderate or severe). Experiencing severe acts of violence was positively associated only with depression and delinquency, whereas experiencing less severe violence was positively associated with depression, PTSD, anxiety, aggressive behavior, and delinquent behavior (Goldner et al., 2015). With respect to witnessing, witnessing severe acts of violence was positively associated with PTSD, and delinquent behavior, whereas witnessing less severe acts was inversely associated with delinquent behavior (Goldner et al., 2015). Studies using factor analysis have also supported the notion that the severity of violence represents a relevant dimension of community violence exposure. Hastings and Kelley (1997) found that three factors based on the severity of exposure represented experiences of violence better than factors that only differentiated between witnessing and direct victimization among a predominately African American sample of adolescents living in high‐crime neighborhoods. Despite these findings, there is a dearth in the literature examining how the severity of community violence experiences influences adolescent adjustment.

Person‐centered analyses such as LCA can model heterogeneity in experiences within a sample by grouping individuals based on similar response patterns (Masyn, 2013). Person‐centered analyses differ from variable‐centered approaches that assume that the relation between variables (independent variable and dependent variable) is the same for each individual (i.e., homogeneous) within a sample (Masyn, 2013). Lanza and Rhoades (2013) described the potential advantages of LCA over traditional dimensional approaches, including the ability to identify subgroups that differ in their patterns across variables. Person‐centered analysis is thus well suited to identify subgroups of adolescents that differ in their patterns of exposure to different forms of community violence. Although several studies have used LCA to examine adolescents' experiences of community violence (e.g., Gaylord‐Harden et al., 2016; Lambert et al., 2021), they have restricted their focus to experiences within a limited time frame (i.e., within the past year), which omits prior experiences that affect adolescent adjustment. Moreover, Gaylord‐Harden et al. (2016) had a small sample (i.e., *N* < 300), which may not have had sufficient power to detect the “true” number of subgroups (Nylund‐Gibson & Choi, 2018). Lambert et al. (2021) examined total scores of witnessing violence and victimization as indicators and did not investigate potential differences in associations between other dimensions of exposure and adjustment.

### The current study

The primary goal of this study was to identify patterns that reflect differences in urban early adolescents' experiences of acts of community violence that differ across multiple characteristics and the extent to which these patterns are related to internalizing symptoms and externalizing behaviors. A secondary goal was to illustrate how person‐centered approaches could be used to identify differences in patterns of risk factors for maladjustment. We conducted an LCA on indicators representing adolescents' reports of their lifetime exposure to violence as victims and witnesses for acts that differed in their degree of severity and the adolescent's relationship with the victim. We hypothesized that variability in adolescents' experiences could be represented by subgroups that differed in their patterns of community violence exposure across different characteristics. Once the subgroups were identified, we formulated specific hypotheses regarding subgroup differences in anxiety symptoms and their frequency of physical aggression. Broadly, we hypothesized that subgroups with the highest levels of violence exposure would have the highest frequencies of physical aggression and levels of anxiety symptoms compared to other subgroups. We also examined the extent to which sex predicted subgroup membership. Several studies have found differences in rates of community violence exposure between boys and girls (e.g., Finkelhor et al., 2015; Javdani et al., 2014; Koposov et al., 2021). However, because studies are inconsistent in their findings these analyses were considered exploratory. Our focus was on a predominately Black sample of adolescents from an urban area. This population is of particular relevance because of their greater risk, compared with White adolescents, for experiencing community violence and other stressful and adverse experiences due to structural inequalities and systemic racism, and negative consequences (Chen et al., 2020; Foster & Brooks‐Gunn, 2009). They are also less likely to have access to and receive services for mental health concerns (Foster & Brooks‐Gunn, 2009). Consequently, it is critical to understand how risk factors, such as community violence exposure, impact adolescent adjustment to refine theories that inform interventions that address mental health needs and prevent maladjustment of adolescents of color residing in neighborhoods with structural inequalities.

## METHODS

### Participants and procedures

This study was based on data collected from two samples of eighth‐grade students attending three public middle schools in Richmond, VA, an urban, southeastern city in the United States. This city has experienced persistent segregation in part due to historical background and movement of economically advantaged individuals to surrounding suburbs. Thus, the city and neighborhoods served by these schools have a high concentration of economically disadvantaged and racially marginalized individuals. The city also had a higher rate of youth violence involvement compared to the national average at the time of data collection. Data were collected from Sample 1 (*N* = 405) in the spring of 1998, 2 years following a cluster‐randomized trial that assigned sixth‐grade classrooms at each of the three schools to intervention or control conditions (see Farrell et al., 2001 for details). The intervention, Responding in Peaceful and Positive Ways (RIPP), consisted of 25 weekly 50‐min sessions focused on social‐cognitive problem‐solving skills to prevent violence (Meyer et al., 2000). Only 34% of Sample 1 had been in a sixth‐grade classroom assigned to the intervention. Data were collected from Sample 2 (*N* = 276) in the spring of 1999, a year following a cluster‐randomized trial that assigned seventh‐grade classrooms at two of the schools to intervention or control conditions (see Farrell et al., 2003 for details). The seventh‐grade intervention consisted of 12 weekly sessions designed to boost the effects of the sixth‐grade intervention (Meyer et al., 2000). Only 38% of Sample 2 had been in classrooms that received the intervention the preceding school year.

The final sample of 643 was obtained by combining the two samples after excluding 38 students (5.6%) whose response pattern met criteria that suggested random responding (Farrell et al., 1991). All students on the class rosters of non‐special education classrooms at the time of data collection were eligible to participate whether or not they had attended the school or participated in earlier waves. Thus, attrition of students over the course of the intervention project was not a concern for the current study as responses are representative of eligible students regardless of prior participation. The project used passive consent whereby parents were notified of the study and given the opportunity to have their adolescents opt out of the study. Students who did not want to participate were told to return blank survey booklets. Data were obtained from approximately 87% of eligible students. All procedures were approved by the IRB of the researchers' University. Participants ranged in age from 12 to 15 years, 58% identified themselves as girls and 42% as boys (other options were not provided), 95% self‐identified as African American, 2% as White, and 0.7% as Latinx.

### Measures

Study staff unaware of prior treatment conditions administered measures to students during homeroom or a class period scheduled for testing. Participants were told that their answers would be kept confidential, and school staff did not handle completed materials.

#### Exposure to violence

Participants self‐reported their lifetime exposure to violence on items from the Children's Report of Exposure to Violence (CREV; Cooley et al., 1995). Items included witnessing violence against a stranger (six items), witnessing violence against a familiar person (six items), and victimization (seven items). *Stranger* was defined as “someone you don't know,” and *familiar person* was defined as “people you already know, such as friends, classmates, relatives, cousins, sisters, brothers, and parents.” Items from the original scale that asked about violence witnessed in the media were excluded. Participants reported how frequently they experienced or witnessed each act on a 4‐point scale ranging from: 1 (*no, never*) to 4 (2*a many times*). Previous studies of the CREV have found good test‐retest reliability, internal consistency, and construct validity (Cooley et al., 1995). Alpha coefficients for the combined samples in the current study were .87 for violence against a stranger, .76 for violence against a familiar person, and .77 for victimization. Items were classified as “severe” based on the stated or implied use of weapons or if they implied threat to life (e.g., stabbed and shot).

#### Anxiety

Participants reported their anxiety symptoms on the Revised Children's Manifest Anxiety (RCMAS; Reynolds & Richmond, 1979). The RCMAS is a 37‐item measure designed to assess current trait and manifest anxiety in youth and adolescents. Items are rated as *yes* or *no*. The RCMAS has three subscales that assess physiological symptoms, worry, and concentration, and a Total Anxiety scale. The alpha for the Total Anxiety Score in the present study was .89.

#### Physical aggression

Physical aggression was assessed using the 7‐item Physical Aggression subscale of the Problem Behavior Frequency Scale (PBFS; Farrell et al., 2000). Confirmatory factor analysis has provided support for four factors: physical aggression, nonphysical aggression, delinquency, and drug use (Farrell et al., 2000). Items on the physical aggression subscale (e.g., “hit or slapped another kid,” “threatened someone with a weapon”) were based on the Youth Risk Survey (Kolbe et al., 1993). Adolescents reported the frequency of engagement in specific acts of physical aggression within the past 30 days on a scale of 1(*never*)to 6 (*20 or more times*). The subscale score was calculated by summing the responses to each item. Cronbach's alpha for the physical aggression subscale in the combined sample was *α* = .87.

### Data analysis plan

All analyses were conducted using Mplus Version 8 (Muthén & Muthén, 2017). We used the maximum likelihood estimation with robust standard errors (MLR), which uses all available data to estimate parameters. We conducted an LCA to identify classes of adolescents with distinct patterns of responses to items on the CREV. Indicators were dichotomized to reflect *never* versus *ever* experiencing each act of violence. The best‐fitting model and optimal number of classes were determined based on reviewing model fit statistics (log likelihood, LL; Akaike information criterion, AIC; Bayesian information criterion, BIC; Bayes factor, BF; approximate correct model probability, cmP), class size consideration, and theory (Masyn, 2013). Likelihood ratio tests, including the Lo–Mendell–Ruben likelihood ratio test (LMR‐LRT), the bootstrap likelihood ratio test (BLRT), and the Vuong‐Lo‐Mendell‐Rubin likelihood ratio test (VLMR‐LRT), were also used to identify the optimal number of classes. Lower AIC and BIC values indicate better fit, and nonsignificant *p *values on the LMR‐LRT, BLRT, and VLMR‐LRT indicate that adding an additional class does not improve model fit (Masyn, 2013). BF values greater than 10 provide strong evidence that Model *k* is the correct model compared to model *k* + 1 (Masyn, 2013). The model with the highest cmP value is considered to be the best fitting model (Masyn, 2013). We placed greater weight on the BF and cmP values when determining the best‐fitting model because they provide a measure of each model's fit relative to the other models under consideration (Masyn, 2013).

After determining the optimal number of subgroups, we examined the resulting patterns and generated hypotheses regarding subgroup differences in physical aggression and anxiety. We then used the manual three‐step Bolck, Croon, and Hagenaars (BCH) approach (Asparouhov & Muthén, 2014) to compare subgroup differences in physical aggression and anxiety while controlling for sex and exposure to the intervention. Physical aggression and anxiety were regressed on a variable indicating each individual's most likely subgroup membership, which takes into account the uncertainty in classification, and dummy coded variables representing sex and intervention condition, with female sex and control conditions representing the reference groups. Pairwise comparisons were used to examine subgroup differences in physical aggression and anxiety. We also used the three‐step approach to regress the variable indicating most likely class membership on variables representing participant sex (dummy coded male/female) to investigate whether sex predicted class membership. To investigate whether exposure to either intervention predicted class most likely class membership, we created dummy coded variables (i.e., Sample 1 intervention condition, Sample 1 control condition, Sample 2 intervention condition) and regressed the variable for most likely class membership on these dummy coded variables. We hypothesized that sample and intervention status would not be a predictor of class membership because the intervention was not designed to reduce exposure to violence.

## RESULTS

### Descriptive statistics

The frequency of endorsement for all CREV items is reported in Table S1. The item most frequently endorsed as having occurred at least once was *Seeing someone you know get beaten up* (80.4% of adolescents reported that they had witnessed this at least once). Over half the sample reported witnessing the following items at least once: *Seen a stranger beaten up, seen a stranger chased/threatened, seen somebody you know beaten up*, and *seen somebody you know chased/threatened*. Less than 10% of the sample reported that they *have been robbed/mugged* at least once. Less than 5% endorsed the highest frequency of exposure on 13 of the 19 items (*Many times)*. Examples of items endorsed *Many times* by less than 5% of the sample are: *Seen a stranger stabbed/killed, seen someone you know robbed/mugged, you have been beaten up, you have been shot/stabbed*. Only 5.1% of adolescents reported that they had never experienced or witnessed any form of community violence.

### Latent class enumeration

LCA was used to test a series of models using 1000 random starts, starting with a one‐class model, and increasing the number of classes until the resulting model was not well identified. This resulted in models specifying between 1 and 8 classes (Table 1). The AIC and adjusted BIC continued to decrease up through the eight‐class model. The BLRT remained significant across all models tested. The five‐class model was supported by the BIC, cmP, and BF. The BF was >100 when comparing the five‐class solution to the four‐class solution, suggesting that the five‐class solution has a higher probability of being the “correct” model. The five‐class model had average posterior class probabilities ranging from .88 to .95, indicating a high degree of class separation and classification precision. The odds of correct class ratios ranged from 24.33 to 149.11, indicating good classification separation and high assignment accuracy (Masyn, 2013). The five‐class solution had high separation of classes (i.e., entropy = .87), and adequate class sizes (i.e., lowest class size was 12%).

**Table 1:** Fit indices for latent class analysis models with dichotomous indicators.

*k*	Par	LL	AIC	BIC	Adj. BIC	VLMR‐LRT (*p*)	LMR‐LRT (*p*)	BLRT (*p*)	Entropy	Smallest class (%)	BF (*K*, *K* + 1)	cmP
1	19	−6766.7	13,571.3	13,657.0	13,596.7	NA	NA	NA	NA	100%	<.001	<.001
2	39	−5669.9	11,417.8	11,593.6	11,469.8	.000	.000	.000	.902	30%	<.001	<.001
3	59	−5424.0	10,966.1	11,232.0	11,044.7	.000	.000	.000	.843	19%	<.001	<.001
4	79	−5268.0	10,694.0	11,050.0	10,799.2	**.001**	**.001**	.000	.851	15%	<.001	<.001
5	99	−5159.0	10,516.0	**10,962.2**	10,647.9	.086	.088	.000	.865	12%	**>100**	**1.000**
6	119	−5103.8	10,445.6	10,982.0	10,604.2	.009	.009	.000	.859	9%	>100	<.001
7	139	−5053.4	10,384.7	11,011.2	10,569.9	.014	.015	.000	.858	5%	>100	<.001
8	159	−5016.0	**10,350.0**	11,066.7	**10,561.9**	.194	.194	.000	.870	4%	NA	<.001
9	Nonconvergence											

*Note*: *N* = 670. *k* = Number of classes; Par = number of parameters*;* LL = log likelihood, BIC = Bayesian information criterion; VLMR‐LRT = Vuong–Lo–Mendell–Rubin likelihood ratio test; LMR‐LRT = Lo–Mendell–Rubin likelihood ratio test; BLRT = Bootstrap likelihood ratio test; BF = Bayes factor; cmP = approximate correct model probability; NA = not applicable; VLMR‐LRT, LMR‐LRT, BLRT, and Entropy not applicable for one‐class models. Values in bold for the AIC, BIC, and adj. BIC indicate the model with the minimum value. Value in bold for the BF indicates the model with the smallest number of classes that is favored over a model with an additional class. Value in bold for the cmP(K) indicates value above .10.

**Figure 1:**
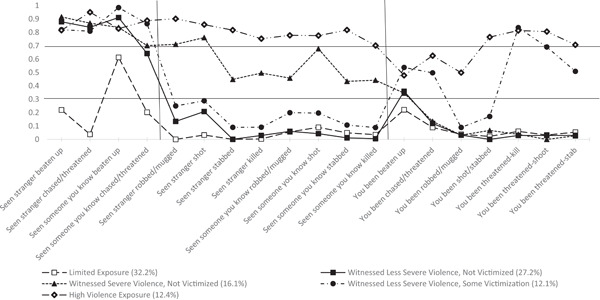
Estimated probability of item endorsement for the binary five‐class solution. Note: Vertical lines separate categories of items representing (from left to right): witnessing less severe acts of violence, witnessing severe acts of violence, and experiencing violence (victimization). Horizontal lines at 0.3 and 0.7 represent cutoffs considered to represent subgroup consistency in not endorsing or endorsing a given item (respectively).

Item endorsement probabilities were examined to determine homogeneity within the classes (see Figure 1). Values above .7 and below .3 indicate homogeneous responses within the subgroup (Masyn, 2013). Within the five‐class model, the most prevalent subgroup (32.2%), labeled *limited exposure*, represented adolescents who tended to report that they had never experienced any of the witnessing or victimization items, with the exception of *seen someone you know beaten up*. The second most prevalent subgroup, labeled *witnessed less severe violence, not victimized* (27.2%), reported witnessing three of the less‐severe acts of community violence (e.g., *seen a stranger get beaten up*), but did not report witnessing or experiencing any of the severe acts of violence. The third subgroup, labeled *witnessed severe violence, not victimized* (16.1%), reported that they had witnessed all four of the less severe items and two of the severe acts of violence, but had not experienced most of the acts of victimization. Within this subgroup, there was less consistency (i.e., probabilities between .30 and .70) in reporting whether they had witnessed the six other items involving severe acts of violence. The fourth subgroup, labeled *witnessed less severe violence, some victimization* (12.1%), was consistent in reporting that they had witnessed the four items involving less severe acts of violence and that they had not witnessed the eight more severe acts. They also reported experiencing one of the acts of victimization (*you have been threatened—killed*), and varied in their reports on four victimization experiences (i.e., each was endorsed by half or more of this group). The final subgroup, labeled *high violence exposure* (12.4%), was consistent in reporting that they had witnessed all different types of community violence against a stranger and a known person, and experienced four of the victimization items.

#### Differences in physical aggression and anxiety symptoms

Once the subgroups had been identified, we developed specific hypotheses regarding differences in their frequency of physical aggression and in anxiety symptoms. These were based on traumatic stress and stress process models of violence exposure (Foster & Brooks‐Gunn, 2009), which suggest that adolescents who are exposed to multiple traumatic events or more severe life‐threatening events are more likely to develop psychopathology compared with other adolescents. We hypothesized that the *high violence exposure* subgroup would have the highest frequency of physical aggression, the *witnessed less severe, some victimization* subgroup would have the second‐highest frequency, the *witnessed less severe, not victimized* and *witnessed severe, not victimized* would have the third‐highest frequencies and be similar to each other, and the *limited exposure* subgroup would have the lowest frequency of physical aggression. In regard to anxiety, we hypothesized that the *high violence exposure* subgroup would report the highest levels, the *witnessed less severe, some victimization* subgroup would report the second‐highest levels, the *witnessed severe, not victimized* subgroup would report the third‐highest levels, the *witnessed less severe, not victimized* subgroup would report the fourth‐highest levels, and the *limited exposure* subgroup would report the lowest levels.

**Figure 2:**
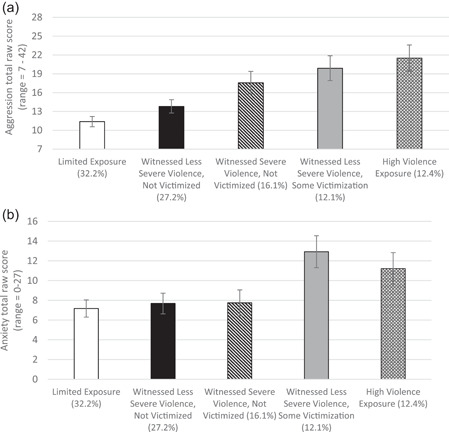
(a) Means of total raw scores and 95% confidence intervals (CIs) of frequency of physical aggression for each subgroup. (b) Means of total raw scores and 95% CIs of frequency of anxiety symptoms for each subgroup. Note: Error bars for each subgroup were calculated based on each subgroup's standard errors.

The three‐step BCH approach was used to compare physical aggression and anxiety across subgroups identified in the five‐class LCA model while controlling for sex and intervention status. Wald tests revealed significant differences across the five subgroups on both physical aggression (*χ* ^2^(4) = 140.7, *p* < .001) and anxiety (*χ* ^2^(4) = 56.3, *p* < .001). Means and 95% confidence intervals (CIs) are presented in Figure 2a,b, and results of pairwise comparisons (d‐coefficients) are reported in Table 2. Pairwise comparisons provided partial support for hypotheses regarding subgroup differences in the frequency of physical aggression. As hypothesized, the *high violence exposure* subgroup reported a higher frequency of physical aggression than all other subgroups (*d*s = 0.50–1.28, *p*s < .01). It did not, however, differ from the *witnessed less severe, some victimization* subgroup. We also found support for our hypothesis that the *witnessed less severe, some victimization* subgroup would report a higher frequency of physical aggression than the *witnessed less severe, not victimized*, and *limited exposure* subgroups (*d*s = 0.75 and 1.07, respectively, *p*s < .001). It did not, however, differ from the *witnessed severe, not victimized* subgroup. The *witnessed severe, not victimized* subgroup reported higher frequencies of physical aggression than the *witnessed less severe, not victimized* subgroup (*d* = 0.46, *p* < .01), which did not support our hypothesis that they would not differ. Finally, as predicted, all subgroups reported higher frequencies of physical aggression than the *limited exposure* subgroup (*d*s = 0.32–1.28, *p*s < .01).

**Table 2:** Effect size estimates (*d*‐coefficients) and 95% confidence intervals represent mean differences in physical aggression and anxiety symptoms across latent class subgroups.

	*d*‐coefficients (95% confidence interval)			
	1	2	3	4
*Frequency of physical aggression*				
1. Limited exposure				
2. Witnessed less severe violence, not victimized	0.32 (0.13–0.50)**			
3. Witnessed severe violence, not victimized	0.78 (0.53–1.03)***	0.46 (0.18–0.74)**		
4. Witnessed less severe, some victimization	1.07 (0.79–1.34)***	0.75 (0.46–1.04)***	0.29 (−0.05 to 0.63)	
5. High violence exposure	1.28 (1.00–1.56)***	0.97 (0.67–1.26)***	0.50 (0.15–0.86)**	0.22 (−0.15 to 0.86)
*Anxiety symptoms*				
1. Limited exposure				
2. Witnessed less severe violence, not victimized	0.09 (−0.14 to 0.32)			
3. Witnessed severe violence, not victimized	0.14 (−0.12 to 0.39)	0.04 (−0.24 to 0.32)		
4. Witnessed less severe, some victimization	0.93 (0.63–1.23)***	0.84 (0.52–1.16)***	0.80 (0.46–1.13)***	
5. High violence exposure	0.72 (0.43–1.02)***	0.63 (0.32–0.94)***	0.59 (0.25–0.93)**	−0.21 (−0.58 to 0.17)

*Note*: Values are *d*‐coefficients representing differences between subgroups identified in the row heading and subgroups listed in each column.

* *p* < .05.

** *p* < .01.

*** *p* < .001.

Partial support was also found for hypothesized subgroup differences in anxiety symptoms. Consistent with our hypothesis, the *high violence exposure* subgroup reported higher levels of anxiety compared with the *witnessed severe, not victimized*, *witnessed less severe, not victimized* subgroup, and *limited exposure* subgroups (*d*s = 0.59–0.72, *p*s < .01). It did not, however, differ from the *witnessed less severe, some victimization* subgroup. As hypothesized, the *witnessed less severe, some victimization* subgroup reported higher levels of anxiety than the *witnessed severe, not victimized*, *witnessed less severe, not victimized*, and *limited exposure* subgroups (*d*s = 0.80–0.93, *p*s < .001). However, the *witnessed severe, not victimized*, *witnessed less severe, not victimized*, and *limited exposure* subgroups did not differ from each other in their reported levels of anxiety.

Finally, we used the three‐step approach to regress the variable indicating most likely class membership on variables representing participant sex, sample, and intervention status. Results of a Wald Test revealed significant sex differences in class membership, *χ* ^2^(4) = 18.01, *p* = .0011. Analyses of ORs by sex within each class revealed one significant difference. Female adolescents were more likely than male adolescents (*p*s .44 and .31, respectively) to be in the limited exposure subgroup, odds ratio = 1.74, 95% CI (1.17, 2.60). Comparison of control samples revealed no significant differences in class membership across the two samples, *χ* ^2^(4) = 8.08, *p* = .089. Comparisons within each sample revealed no significant differences between those who were exposed to the RIPP intervention in either the sixth grade, *χ* ^2^(4) = 2.84, *p* = .648, or seventh grade, *χ* ^2^(4) = 1.08, *p* = .898.

## DISCUSSION

The primary goal of this study was to identify patterns of community violence exposure as victims or witnesses that account for variations in the experiences of Black adolescents in urban areas, and to investigate their relations to adjustment including internalizing symptoms and externalizing behavior. Analyses were conducted on items representing acts of community violence and victimization that differed based on the severity of the act (e.g., being chased or threatened vs. being robbed or mugged) and the identity of the victim (i.e., themselves, someone they knew, a stranger). Only 5% of adolescents reported no experiences of community violence exposure, which underscores the importance of understanding consequences of exposure. Even adolescents in the *limited exposure* subgroup had a probability greater than .50 of reporting that they had witnessed someone they know beaten up. Results of an LCA identified five subgroups of adolescents that differed in their patterns across these characteristics of exposure. They also partially supported the hypothesis that subgroups with different patterns of exposure would differ in their reported frequencies of aggressive behavior and anxiety symptoms. A key finding was that not all characteristics are salient in distinguishing between patterns of exposure or examining associations with adjustment. Overall, the findings suggest that exposure to community violence, regardless of the severity of the act or the adolescents' relation to the victim, impacts adolescents' mental health, which highlights the importance of preventing community violence exposure.

The number and characteristics of the subgroups identified in this study differed from the few previous studies that have conducted the person‐centered analysis. In particular, five subgroups were identified in this study versus three in a prior study by Gaylord‐Harden et al. (2016). This difference could be due to the larger sample size in the present study (*N* = 670 vs. 241), or its inclusion of a greater number and variety of items (19 vs. 10) and experiences of violence. Some items included in the study by Gaylord‐Harden et al. were actions by others that did not involve a clear threat (e.g., *saw someone with a weapon*). Their measure also included items that lacked specificity. For example, items such as *someone close to you was shot or attacked* could mean a close proximity or close relationship. This is an important distinction. Violence that occurs in close proximity could indicate that the adolescent was also in danger, whereas violence that occurs to someone with whom an adolescent has a close relationship which could disrupt the adolescents' support system (Foster & Brooks‐Gunn, 2009). Gaylord‐Harden et al. identified one subgroup characterized by “high” and one characterized by “low” levels of total community violence exposure (i.e., witnessing and experiencing violence), and one subgroup with high levels of only experiencing violence. In contrast, the current study identified subgroups that differed as a function of the severity of violence and based on witnessing versus experiencing violence. We did not identify any subgroups who reported experiencing, but not witnessing violence. These patterns reflect a wider variability in adolescents' exposure to violence than would be represented by a single continuous indicator. Studies that use one or two indicators to quantify violence exposure (e.g., Lambert et al., 2021) or variable‐centered approaches could miss patterns of exposure that result in differences in adolescent adjustment. Overall, these findings underscore the importance of assessing a wide variety of adolescents' experiences within their community to understand the impact of their environment on adjustment.

The findings highlight the importance of differentiating between witnessing versus experiencing violence, particularly when examining associations with anxiety symptoms. We identified two subgroups of adolescents who reported witnessing violence, but not experiencing victimization; two subgroups who reported both witnessing violence and victimization; and a subgroup who reported little, if any exposure through witnessing or victimization. The pattern of results revealed significantly higher levels of anxiety for the two groups that reported victimization compared with the three groups that did not (i.e., *d*s = 0.59–0.93). There were not, however, any significant differences in anxiety between the two groups reporting victimization or among the three groups that did not. This suggests that, at least for anxiety symptoms, experiencing victimization is a more salient factor than witnessing regardless of the severity of violence witnessed. These results are consistent with a meta‐analysis by Fowler et al. (2009) and support traumatic stress theories that suggest that victimization rather than witnessing violence results in traumatic stress and difficulties with emotion regulation. These findings are also consistent with the trauma literature, which suggests that victimization poses a greater threat to an individual and consequently has a greater impact on adjustment (Kennedy & Ceballo, 2014).

The absence of differences in anxiety across subgroups that differed in the severity of violence they witnessed differs from prior studies that have found that witnessing more versus less severe acts are associated with higher levels of internalizing symptoms (e.g., Aisenberg et al., 2008; Goldner et al., 2015). This difference in findings could be due to differences in how the measures categorized the severity of items. For example, Aisenberg et al. (2008) determined “severity” of exposure by asking participants which acts of violence they experienced that were *most bothersome*, and considering the length of court sentence for a given act of violence. They found that the acts of violence that adolescents reported as *most bothersome* were often not the most objectively severe acts. Moreover, they investigated symptoms of PTSD, rather than anxiety as their outcome. Further, findings from Aisenberg et al. (2008) and Goldner et al. (2015) examined the variability in adjustment accounted for by different constructs of violence exposure, whereas LCA focuses on how the patterns of exposure observed in a sample are associated with differences in adjustment. These approaches answer different questions, which could explain differences in findings regarding the severity of exposure.

Analysis of subgroup differences in the frequency of physical aggression revealed differences related to both the type of exposure and the severity of acts of violence witnessed. Among the three subgroups that did not report victimization, the frequency of physical aggression was highest for the subgroup that reported witnessing severe acts of violence, followed by the subgroup that reported witnessing some less severe acts of violence, and the subgroup that had limited exposure to witnessing. In contrast, the two subgroups that reported victimization reported the highest frequency of physical aggression, but did not differ from each other despite differing dramatically in the number of severe acts they reported witnessing (one reported no severe acts, the other endorsed all of them). This suggests that victimization experiences have an additive effect that compounds the effects of witnessing less severe acts of violence. These findings are consistent with the conclusions of a meta‐analysis by Fowler et al. (2009). Findings from the current study are also consistent with Goldner et al. (2015) who found that witnessing severe violence, compared with witnessing less severe violence, was more strongly correlated with externalizing behaviors (i.e., aggressive behavior and delinquency).

Although prior studies have found that adolescents' adjustment varied depending on the degree of familiarity with a victim of violence (Lambert et al., 2012; Ward et al., 2001), the current study did not identify subgroups that differed based on their familiarity with the victim. This may reflect the measures, which used a broad definition of someone familiar. Results may have differed if the definition had been more narrowly focused on family members or other close relationships. Lambert et al. (2012) found that witnessing violence against a close friend or family member was associated with symptoms of anxiety and depression, and witnessing violence against an acquaintance and stranger were not. Thus, we might have found different patterns of exposure if our definition of familiarity was more limited.

The use of a person‐centered approach provided a nuanced perspective on adolescents' exposure to witnessing and experiencing community violence compared with the majority of prior studies that have used a variable‐centered approach that represents experiences along a continuum. Whereas some prior studies using factor analysis to examine the structure of measures of exposure to violence have found support for factors representing witnessing violence versus victimization (e.g., Martin et al., 2013; van Dulmen et al., 2008), others have identified factors that vary across characteristics such as severity (Hastings & Kelley, 1997), and familiarity with the victim (Cooley et al., 1995). In most cases the resulting factors are moderately to highly correlated, suggesting that higher frequencies of some experiences are associated with high frequencies of other experiences. However, this is not the case for all adolescents as evident by two subgroups that reported high frequencies of witnessing violence but little to no experiences of victimization identified in the current study. The person‐centered approach was particularly valuable when looking at differences in adjustment and suggests that frequency of physical aggression and symptoms of anxiety differ in ways that might not be captured by associations with one or two scores representing violence exposure. In sum, person‐centered approaches can identify subgroups that represent the covariation across the indicators.

Community violence exposure represents one environmental risk factor for adverse outcomes experienced by adolescents residing in communities that are under‐resourced and economically disadvantaged (Foster & Brooks‐Gunn, 2009). These adolescents often encounter multiple risk factors, but differ in their specific experiences. Thus, there is heterogeneity in experiences and risk factors within a community of adolescents. Understanding the ways in which an adolescents' environment impacts their adjustment is critical for refining both theory and intervention efforts. Person‐centered approaches could be useful in identifying subgroups of adolescents with different patterns across a wider range of risk factors (e.g., parental factors, peer factors, etc.). Understanding heterogeneity in risk for maladjustment within a community could then be used to enhance community‐level interventions by using patterns in risk factors, rather than pre‐determined criteria, to inform the allocation of resources (e.g., providing adult support for adolescents with low parental monitoring) and tailoring of interventions to meet the needs of identified subgroups. Identifying subgroups based on multiple dimensions of risk may help maximize intervention effects compared to approaches that group based on single pre‐determined characteristics (Lanza & Rhoades, 2013).

Another area of intervention research that might benefit from person‐centered approaches is investigating mechanisms that underly relations between witnessing community violence and physical aggression and anxiety. Interventions often target malleable underlying mechanisms to prevent maladjustment. Because subgroups identified in the current study differed in both their experiences and their symptomatology, they may also differ in the mechanisms underlying these relations. Lambert et al. (2010) found that adolescents in a subgroup with high levels of violence exposure exhibited more impulsive behavior, one factor that has been associated with aggressive behavior, compared to adolescents in a subgroup with lower levels of violence exposure. In the current study, it is possible that mechanisms that underly associations between experiences of victimization and physical aggression and anxiety differ from those that underly associations between witnessing violence and physical aggression. Thus, adolescents may differ in the extent to which they benefit from different interventions based on which mechanisms the interventions target. For example, the RIPP intervention implemented in the larger study targeted adolescents' beliefs about aggression and conflict problem‐solving skills to prevent aggressive behavior (Meyer et al., 2000). This intervention could be beneficial among adolescents for whom beliefs about aggression and problem‐solving skills are thought to be driving engagement in aggressive behavior. In contrast, adolescents with experiences of victimization and who are experiencing distress (e.g., anxiety symptoms), such as those in the *high violence exposure* and the *witnessed less severe violence, some victimization* subgroups, may benefit from trauma‐informed interventions that target both underlying mechanisms of behavior problems (e.g., core beliefs) and symptoms of trauma (e.g., flashbacks and emotional distress). Future studies should use person‐centered approaches to identify potential subgroup differences in mechanisms that underly relations between violence exposure and maladjustment to inform intervention tailoring and selection for different subgroups of youth.

### Limitations

Although this study addressed several gaps in the literature on community violence exposure, there are several limitations that need to be considered. This study relied on adolescent self‐report, which could be biased or inaccurate. For example, youth might under‐report their aggressive behavior, and prior studies have found low‐to‐moderate agreement across informants for aggression (e.g., Farrell et al., 2016). However, adolescents may be better informants than their caregivers or teachers on certain measures, particularly behavioral measures or experiences that occur in the community. Parents and teachers are less likely to see adolescents engaging in behaviors outside of the home and school. This is reported by studies indicating that parents underreport their adolescents' frequency of violence exposure (Martinez & Richters, 1993). This study examined cross‐sectional data, which precludes conclusions regarding the direction of relations between community violence exposure and adolescent anxiety and physical aggression. However, a cross‐sectional design was sufficient for the primary aim of this study, which was to identify subgroups of adolescents that differed in their patterns of experiencing community violence and how these patterns are related to their adjustment. Because the sample was urban and primarily included low‐income, Black adolescents, the findings might not generalize to adolescents of other races, ethnicities, or contexts (i.e., rural or suburban contexts). Although previous research suggests that community violence might have different effects on other symptoms of distress (e.g., PTSD, depression) the original study only assessed anxiety symptoms. Additionally, our construct of familiarity was dichotomous (i.e., whether or not the victim of violence was a stranger). There are different levels of familiarity with another individual (i.e., acquaintance, friend, and family member), and prior studies indicate that the level of familiarity differentially impacts relations between community violence exposure and mental health symptoms (e.g., Lambert et al., 2012). Thus, additional nuances in the relation adolescents may have with a victim of community violence, or between a victim and a perpetrator, were not captured in this study. There are, of course, other areas of research that focus on victimization by individuals of significant relation to the victim of violence (e.g., intimate partner violence and abuse by caregivers). A final limitation is the age of the data used in this study. The data were collected over 20 years ago. Advances in media and access to information could increase the rate at which youth are indirectly exposed to community violence (e.g., through social media platforms), which could also impact mental health. However, recent studies indicate that adolescents continue to experience the same forms of in‐person violence experienced by adolescents in the current study (e.g., Chen et al., 2020). Thus, results are likely still generalizable to current adolescents.

## CONCLUSION

The findings of this study addressed several gaps in the literature regarding the ways in which adolescents' experiences of community violence differ across characteristics, specifically what patterns exist and how these patterns are associated with differences in adjustment. Findings have implications for both the measurement of risk factors and intervention efforts. Our findings highlight the utility of subgroup analyses in identifying patterns in adolescents' exposure to acts of community violence beyond a simple correlation coefficient. These patterns would not have been identified by variable‐centered approaches. Adolescents residing in communities that have historically encountered structural inequalities, high rates of violence, and have low access to resources often experience a number of stressors and risk factors for maladjustment in addition to violence exposure (Foster & Brooks‐Gunn, 2009). Person‐centered analysis could be used to investigate patterns across a wider range of experiences and risk factors (e.g., discrimination, adverse life experiences such as death of a parent) and their associations with adolescent adjustment. Finally, the high prevalence of violence exposure in this study (i.e., 95%) suggests that community violence exposure is a risk factor for most adolescents, and those who were exposed had an increased risk for maladjustment. This underscores the need for interventions that target structural causes of community violence, such as community‐based violence prevention programs, to reduce rates of exposure. Person‐centered approaches within universal programs could be used to identify patterns of risk associated with the highest levels of maladjustment, which could inform the allocation of resources to bolster the effects of programs (e.g., which youth are in most need of mental health care). Additionally, increasing community‐level protective factors, such as mentoring programs, after‐school programs, and mental health access, may also help reduce negative consequences of adjustment for many adolescents.

## CONFLICTS OF INTEREST

The authors declare no conflicts of interest.

## Supporting information

Supporting information.
