# Supplementary material for: Patterns of community violence exposure among urban adolescents and their associations with adjustment
Source: Am J Community Psychol. 2022 Apr 28;70(3-4):265–77. doi: 10.1002/ajcp.12598 (PMC9613812; doi:10.1002/ajcp.12598)
Supplement: Supplementary file 1 — Supporting information. [file AJCP-70-265-s001.docx]

**Appendix A.**

Table S1

Percentages of Participants Endorsement of Each Category for Items on the Children’s Report of Exposure to Violence (CREV)

|  | No never | One time | A few times | Many times |
| --- | --- | --- | --- | --- |
| Seen a stranger beaten up | 33.9 | 22.2 | 25.4 | 18.5 |
| Seen a stranger chased/threatened | 40.0 | 22.1 | 23.2 | 14.7 |
| Seen a stranger robbed/mugged | 70.3 | 12.8 | 11.0 | 5.9 |
| Seen a stranger shot | 66.6 | 18.4 | 9.3 | 5.7 |
| Seen a stranger stabbed | 81.5 | 8.3 | 5.9 | 4.2 |
| Seen a stranger killed | 80.4 | 10.2 | 5.0 | 4.4 |
| Seen somebody you know beaten up | 19.6 | 21.6 | 38.4 | 20.4 |
| Seen somebody you know chased/threatened | 42.9 | 22.3 | 23.5 | 11.4 |
| Seen somebody you know robbed/mugged | 77.2 | 13.1 | 6.6 | 3.1 |
| Seen somebody you know shot | 72.7 | 13.4 | 9.3 | 4.7 |
| Seen somebody you know stabbed | 79.7 | 10.8 | 5.6 | 3.8 |
| Seen somebody you know killed | 81.7 | 9.2 | 5.9 | 3.2 |
| You have been beaten up | 64.8 | 23.2 | 10.2 | 1.8 |
| You have been chased/threatened | 77.7 | 14.1 | 5.9 | 2.3 |
| You have been robbed/mugged | 90.2 | 7.3 | 2.4 | 0.0 |
| You have been shot/stabbed | 86.6 | 9.1 | 3.0 | 1.2 |
| You have been threatened-kill | 76.0 | 13.9 | 7.5 | 2.7 |
| You have been threatened-shoot | 79.2 | 12.0 | 6.2 | 2.6 |
| You have been threatened-stab | 81.7 | 11.1 | 4.8 | 2.4 |

*Note.* *N* = 670. Order of items is based on how the appear on the CREV
